# Supplementary material for: Gene Expression and Chondrogenic Potential of Cartilage Cells: Osteoarthritis Grade Differences
Source: Int J Mol Sci. 2022 Sep 13;23(18):10610. doi: 10.3390/ijms231810610 (PMC9504485; doi:10.3390/ijms231810610)
Supplement: Supplementary file 1 [file ijms-23-10610-s001.zip › ijms-1869036-supplementary.pdf]

Table S1. Statistical differences in the expression of MPC, chondrocyte and hypertrophic chondrocyte markers between BM - MSC commercial cell line and OA grades (Mild, Moderate, Severe).

Markers	BM-MSC vs. MIld	BM-MSC vs. Moderate	BM-MSC vs. Severe	Mild vs. Moder-ate	Mild vs. Severe	Moderate vs. Se-vere
**MPC markers**						
CD105	ns	ns	p= 0.0105, *	ns	Ns	p= 0.0245, *
CD166	p= 0.0480, *	p= 0.0037, **	p= 0.0014, **	ns	Ns	ns
Notch – 1	ns	p= 0.0417, *	ns	ns	Ns	ns
Sox – 9	p= 0.0043, **	p= 0.0010, **	p= 0.0015, **	p= 0.0106 **↑	p= 0.0041, **↑	ns
**Chondrocyte markers**						
Col II	p= 0.0238, *	p= 0.0081, **	p= 0.0077, **	ns	Ns	ns
Acan	p= 0.0025, **	p= 0.0010, **	p= 0.0009, ***	ns	Ns	p= 0.0282, *
**Hypertrophic chondrocyte markers**						
Col I	ns	ns	ns	ns	Ns	ns
MMP13	p= 0.0095, **	p= 0.0032, **	p= 0.0032, **	ns	Ns	ns
ALPL	p= 0.0101, *	p= 0.0081, **	p= 0.0056, **	ns	Ns	ns

Table S2. Statistical differences in the expression of MPC, chondrocyte and hypertrophic chondrocyte markers in between D0 and D14 in mild OA derived cells.

**MILD OA**			
**Markers**	**D0 vs. D14**	**D0 vs. D35**	**D14 vs. D35**
**MPC markers**			
CD105	p= 0.0079, **	ns	p= 0.0079, **
CD166	p= 0.0079, **	ns	p= 0.0079, **
Notch – 1	p= 0.0159, *	ns	p= 0.0079, **
Sox – 9	p= 0.0357, *	ns	p= 0.0159, *
**Chondrocyte markers**			
Col II	ns	p= 0.0357, *	p= 0.0317, *
Acan	p= 0.0159, *	p= 0.0159, *	p= 0.0159, *
**Hypertrophic chondrocyte marker**			
Col I	ns	ns	ns

Table S3. Statistical differences in the expression of MPC, chondrocyte and hypertrophic chondrocyte markers in between D0 and D14 in moderate OA derived cells.

**MODERATE OA**			
**Markers**	**D0 vs. D14**	**D0 vs. D35**	**D14 vs. D35**
**MPC markers**			
CD105	p= 0.0002, ***	p= 0.0159, *	p< 0.0001, ** *
CD166	p < 0.0001, ***	p < 0.0001, ***	p= 0.0002, ** *
Notch – 1	p < 0.0001, ***	p < 0.0001, ***	p= 0.0494, *
Sox – 9	ns	ns	p= 0.0098, **
**Chondrocyte markers**			
Col II	p = 0.0003, ***	p= 0.0391, *	p< 0.0001, ** *
Acan	ns	p= 0.0146, *	p= 0.0186, *
**Hypertrophic chondrocyte marker**			
Col I	ns	**p < 0.0001, *** **	**p < 0.0001, *** **

Table S4. Statistical differences in the expression of MPC, chondrocyte and hypertrophic chondrocyte markers in between D0 and D14 in severe OA derived cells.

**SEVERE OA**			
**Markers**	**D0 vs. D14**	**D0 vs. D35**	**D14 vs. D35**
**MSC markers**			
CD105	p= 0.0005, ***	ns	p= 0.0008, ** *
CD166	p= 0.0028, **	p= 0.0002, ***	p= 0.0076, **
Notch – 1	ns	ns	p< 0.0001, ** *
Sox – 9	ns	ns	ns
**Chondrocyte markers**			
Col II	p < 0.0001, ***	p= 0.0004, ***	ns
Acan	p < 0.0001, ***	p < 0.0001, ***	p= 0.0455, *
**Hypertrophic chondrocyte marker**			
Col I	ns	ns	**ns**
